# Supplementary figures and images for: Impaired functions of human monocyte-derived dendritic cells and induction of regulatory T cells by pathogenic Leptospira
Source: PLoS Negl Trop Dis. 2023 Nov 20;17(11):e0011781. doi: 10.1371/journal.pntd.0011781 (PMC10695387; doi:10.1371/journal.pntd.0011781)

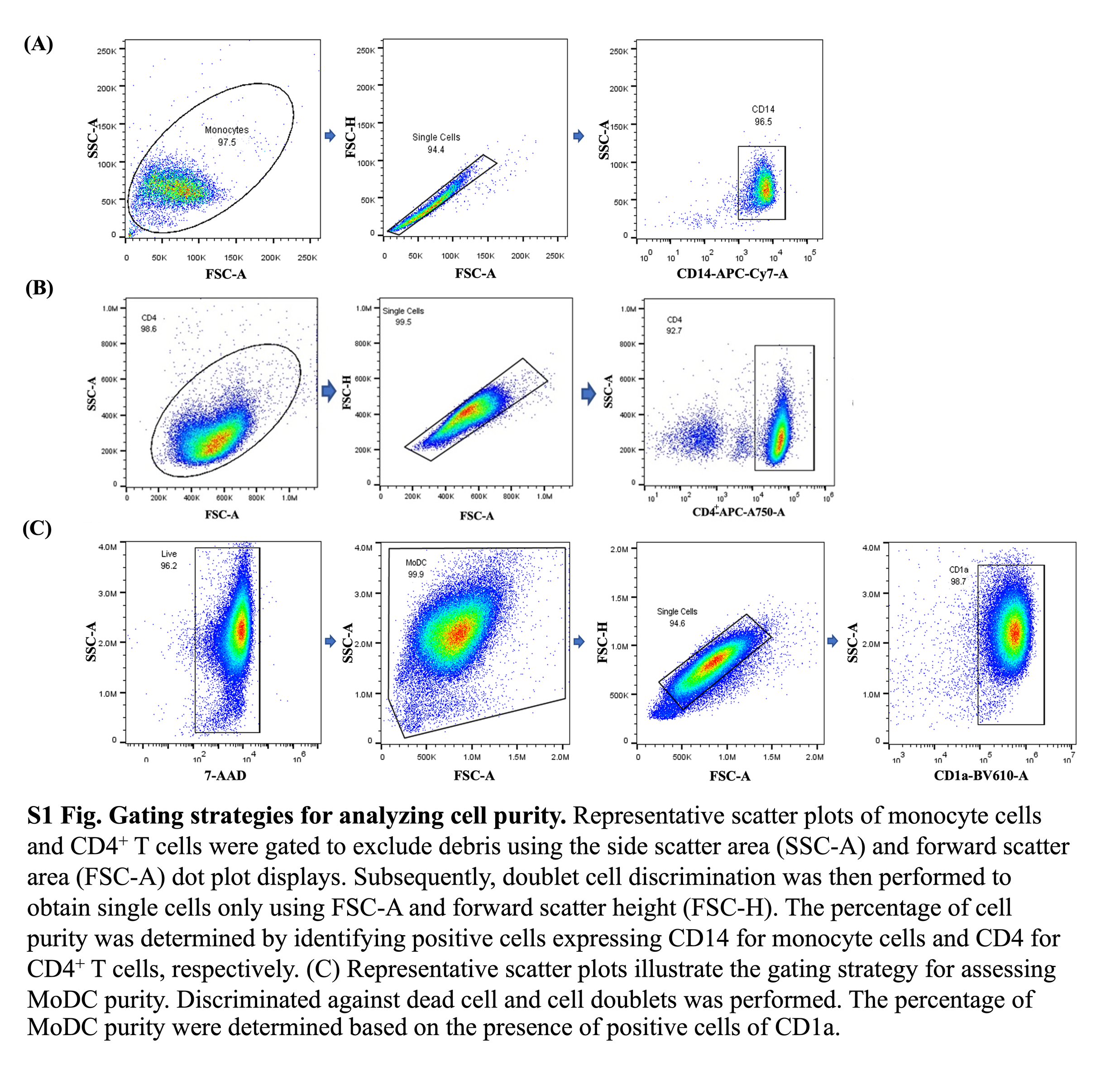

Supplement: S1 Fig — (TIF) [file pntd.0011781.s001.tif]

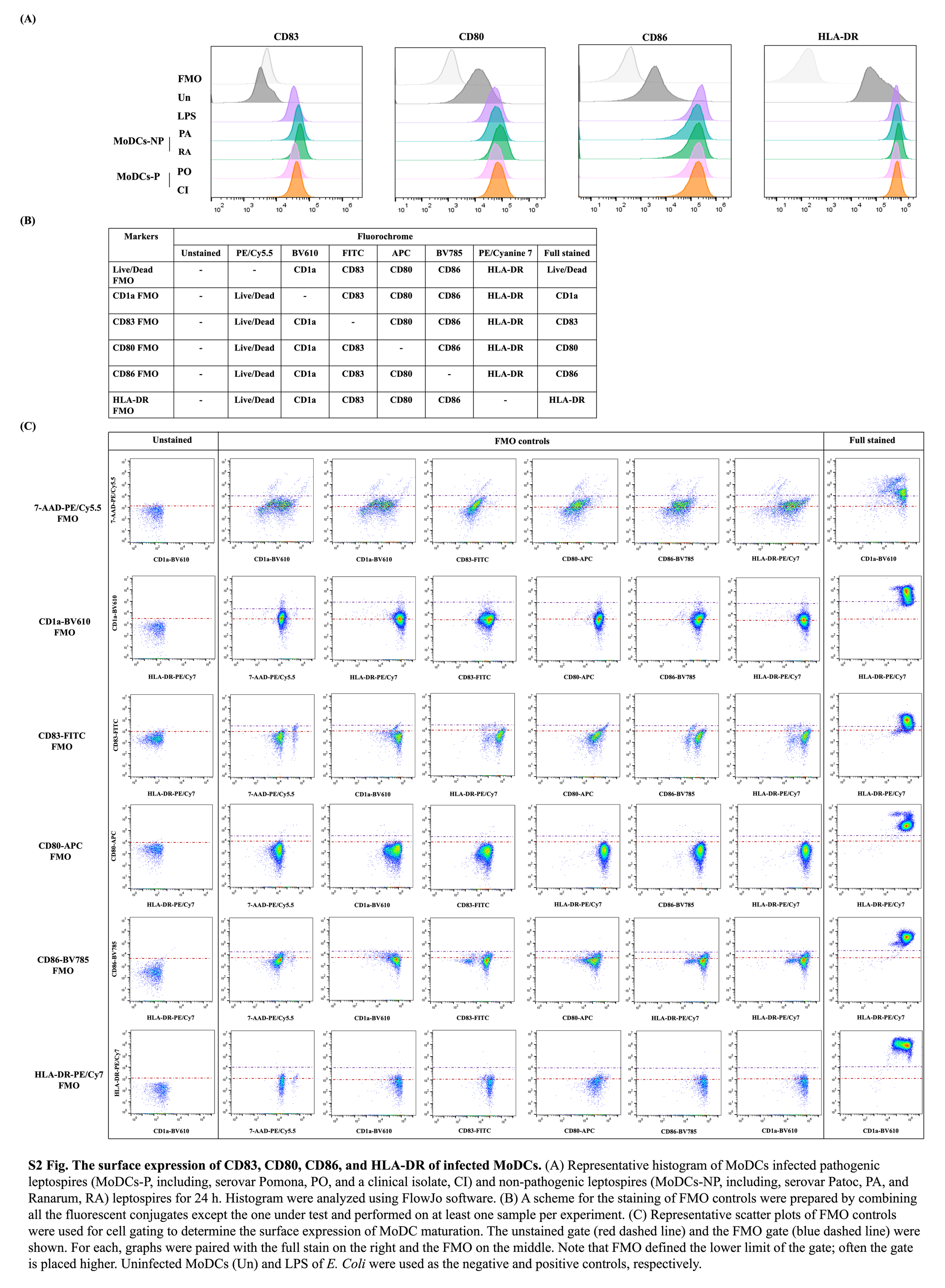

Supplement: S2 Fig — (TIF) [file pntd.0011781.s002.tif]

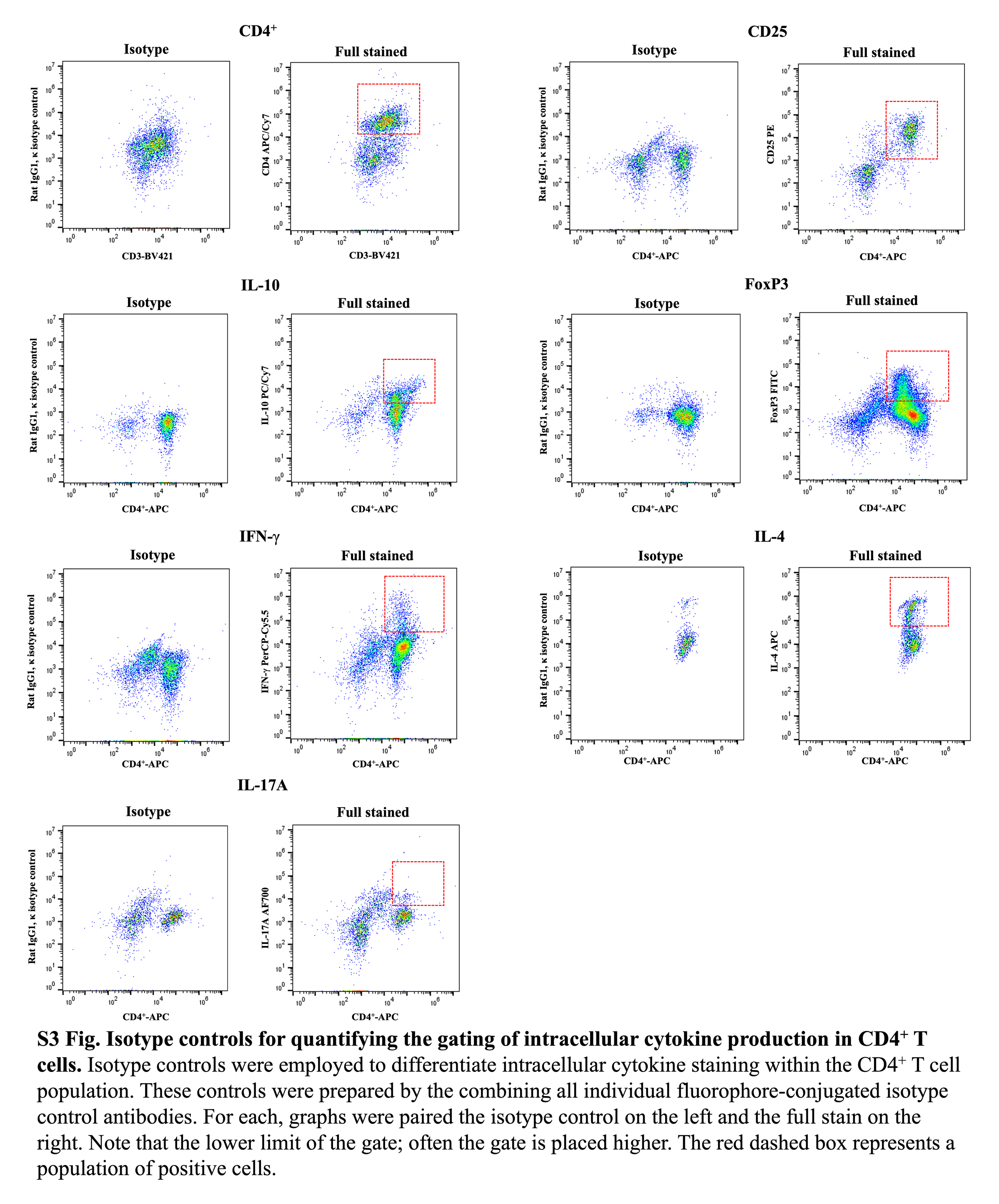

Supplement: S3 Fig — (TIF) [file pntd.0011781.s003.tif]

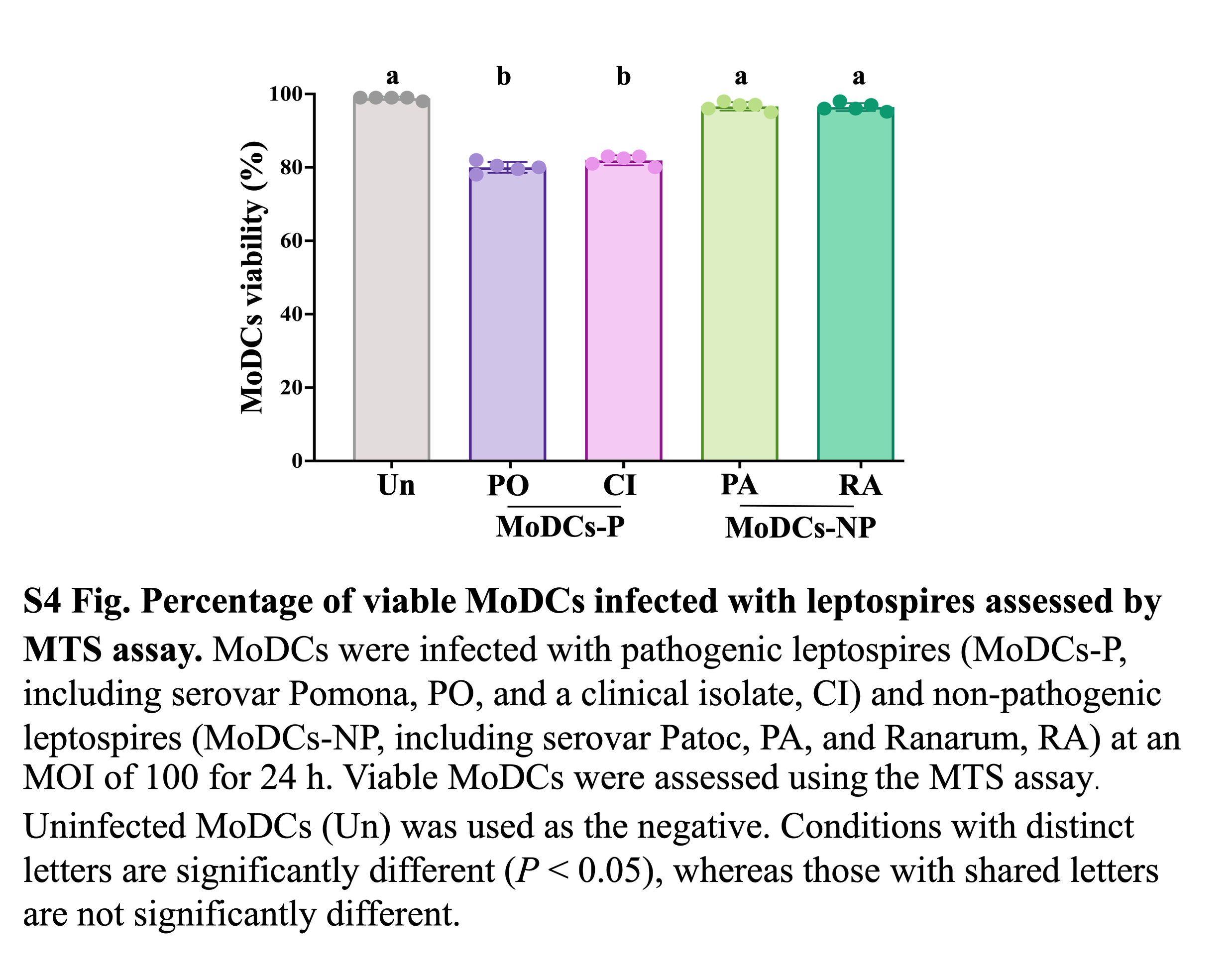

Supplement: S4 Fig — (TIF) [file pntd.0011781.s004.tif]

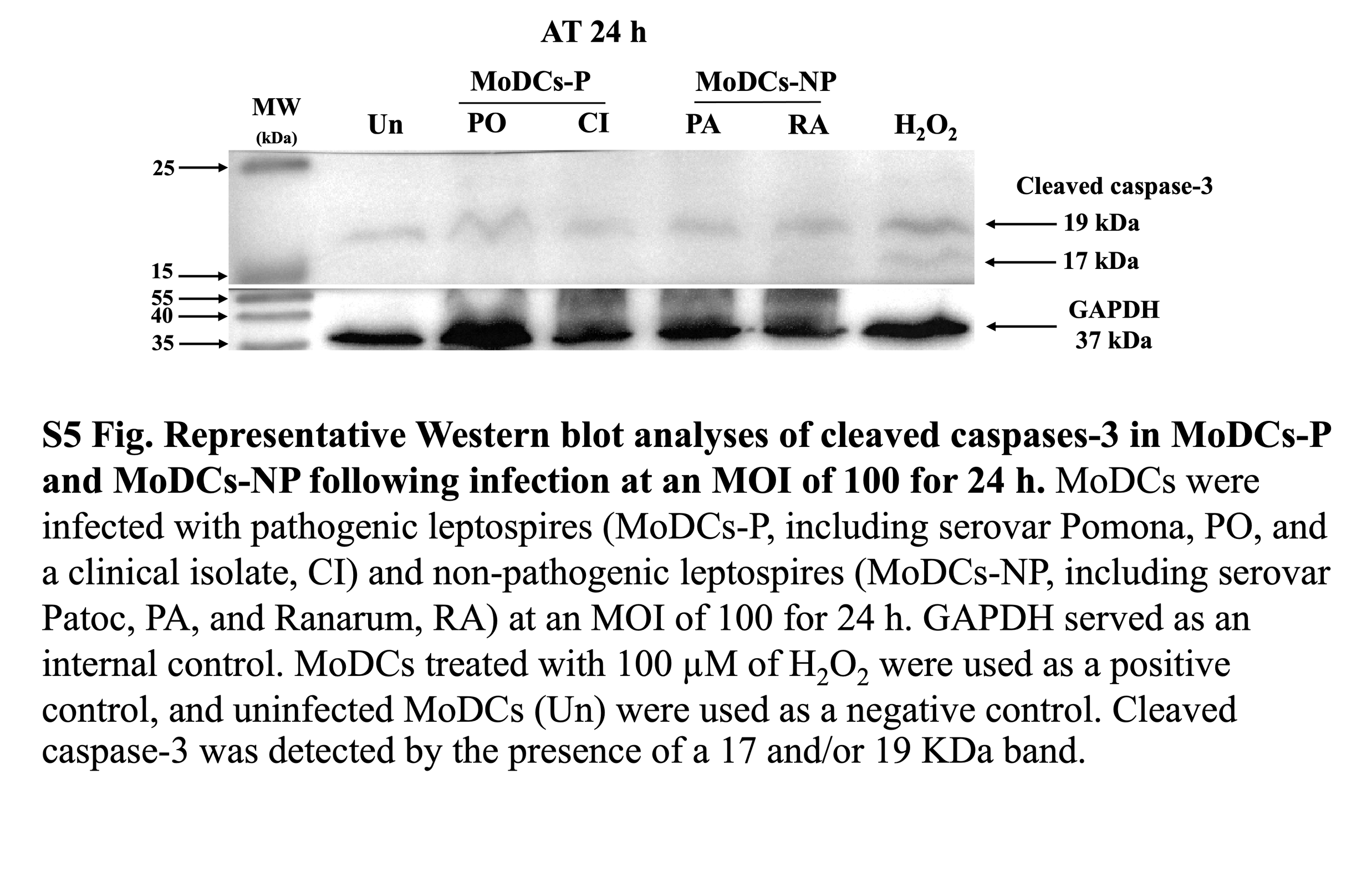

Supplement: S5 Fig — (TIF) [file pntd.0011781.s005.tif]

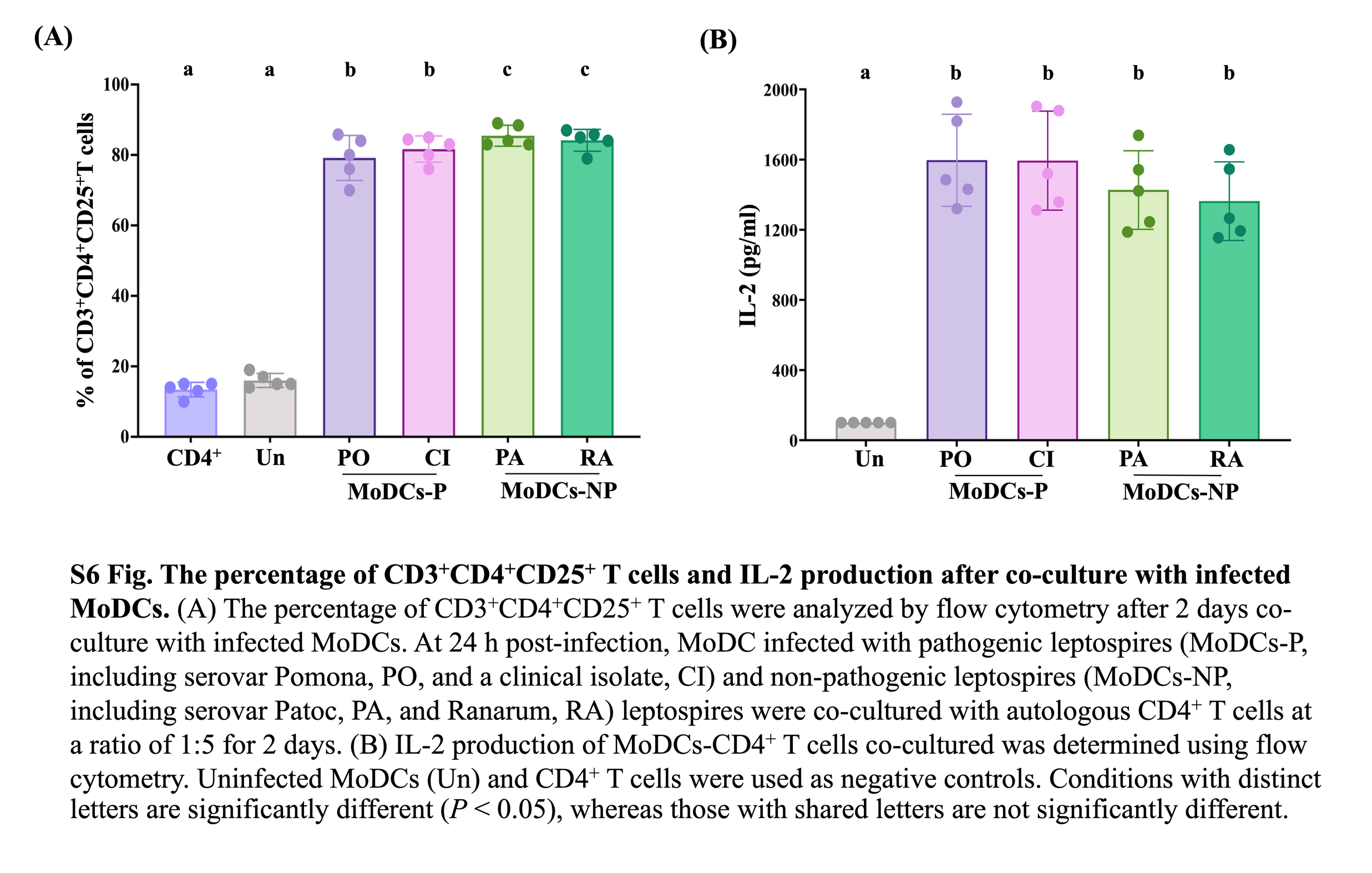

Supplement: S6 Fig — (TIF) [file pntd.0011781.s006.tif]
