# Supplementary material for: Impaired functions of human monocyte-derived dendritic cells and induction of regulatory T cells by pathogenic Leptospira
Source: PLoS Negl Trop Dis. 2023 Nov 20;17(11):e0011781. doi: 10.1371/journal.pntd.0011781 (PMC10695387; doi:10.1371/journal.pntd.0011781)
Supplement: Attachment — Submitted filename: Comments to Manuscript PNTD-00469.docx [file pntd.0011781.s007.docx]

Comments to Manuscript PNTD-D-23-00469

Krangvichian and his co-authors in this study describe dampening of dendritic cell function and induction of a regulatory T cell function during Leptospiral pathogenesis. The work is commendable as it explores different immune cell networking process involved in Leptospiral transmission and dissemination. The group also described the difference between pathogen versus nonpathogenic *Leptospira* sp. There are, however, several parts of the study that needs further clarification or modifications to conclude current observation. Following are few points:

**Major points:**

1. This study utilized monocyte derived dendritic cells to describe the leptospiral pathogenesis. It would be great if observed phenomena can be correlated through an in vivo study.
2. Line102-104, authors are requested to provide justification why both interferon gamma production and increase of CD4+ cells were induced by the nonpathogenic strain. Does nonpathogenic *Leptospira* sp. Infection require induction of an adaptive immunity, or it is efficiently cleared by the innate immune cells?
3. Line120: Why it needs an aerobic condition to grow? If so, how it was grown in culture in aerobic condition?
4. Line141: on day7, the immature DCs were stimulated with leptospires (100 MOI). When this DC maturation occurs in vitro? How is their maturation determined?
5. For the whole study, author needs to provide detailed method of generation of moDC in vitro and how the replated in 24 well plate for leptospiral stimulation. What cellular detachment technique were used during this process? Did the author stimulate the cells with *Leptospira* in complete medium with antibiotics? Details of the methods will be helpful to evaluate the observed phenomena in more details.
6. Line206-207: Cells incubated at 4 degree C used as a negative control. Please provide detail of doing so. For phagocytosis assay, CFSE stained bacteria were used. How could the author determine the bacterial multiplication with CFSE?
7. The IL10 production was less with pathogenic strain whereas the intracellular cytokine staining of CD4+ cells showed opposite phenomenon. The authors are requested to justify the observed fact which is contradictory. Author could check the cytokines in the patient sera for all the cytokines and correlate the observed findings if it matches with the moDC/CD4+cells data.
8. Line508-510: The expression levels of CD83 were reduced a little than that of the nonpathogens whereas the CD86 expression remain similar for all the infected groups. Based on that it is hard to conclude such process without indetail investigation.
9. The authors did not check the other cell death pathway. May be other cell death processes are important during leptospiral or any infection. Authors could cite the recent articles published (PMID: 35392072, 33221042) in line with this process and add a discussion on how cell death influences the inflammation during infections.

Overall, the manuscript is in great shape and will provide knowledge in the field. This could be considered for publication after potential comments are addressed.
