# Supplementary material for: Impaired functions of human monocyte-derived dendritic cells and induction of regulatory T cells by pathogenic Leptospira
Source: PLoS Negl Trop Dis. 2023 Nov 20;17(11):e0011781. doi: 10.1371/journal.pntd.0011781 (PMC10695387; doi:10.1371/journal.pntd.0011781)
Supplement: Attachment — Submitted filename: Response to reviewers.docx [file pntd.0011781.s008.docx]

Faculty of Medicine, Chulalongkorn University

1873 Rama IV Road, Pathumwan

Bangkok, 10330 THAILAND

Kanitha.Pa@chula.ac.th; Kpatarakul@gmail.com

September 19, 2023

Dear Elsio Wunder Jr,

Thank you for providing us with the opportunity to revise our manuscript, Manuscript number: PNTD-D-23-00469, titled “Impaired functions of human monocyte-derived dendritic cells and induction of regulatory T cells: a potential mechanism of immune evasion of pathogenic *Leptospira*”

We greatly appreciate the invaluable suggestions, and we have made the necessary revisions based on the reviewers’ advice. Please note that the line numbers in our response refer to the lines of the revised manuscripts.

***As an editor mention:*** *While all the reviewers acknowledged the importance and innovative aspects of your work, they raised concerns with writing clarity, data interpretation and methodological approaches of your manuscript. The reviewers requested additional experiments to validate some of your conclusions, particularly experiments related to phagocytosis and apoptosis studies. Please, carefully address all the comments listed by the reviewers before submitting a revised version of your manuscript and let the journal know if you will need additional time to prepare your revised manuscript.*

**Response:** Thank you for your feedback on our manuscript. We have carefully reviewed all the comments provided by the reviewers and have identified three main areas of concern that we are addressing in the revised version of our manuscript.

Firstly, we acknowledge the importance of improving the writing clarity of our manuscript. We have extensively revised the text to enhance grammar, sentence structure, and overall coherence, ensuring effective communication of our research findings. To facilitate your review, we have referenced specific line numbers in this response letter that correspond to the revised manuscript.

Secondly, we concur with Reviewer #3’s viewpoint (*removing parts that are not essential for the purpose of this study (transcriptomic and phagocytosis)* regarding the removal of non-essential components from our study, specifically the phagocytosis assay*.* We agree that quantifying leptospiral phagocytosis by MoDC is not central to our objectives, and the determination of the intracellular location of leptospires does not definitely indicate phagocytosis. Therefore, we have removed the method and result of the phagocytosis section from the revised manuscript. This removal does not change the main conclusion of our manuscript.

Lastly, we greatly value the reviewers’ concern regarding the determination of MoDC apoptosis. To address this issue, we have conducted additional experiments involving varying MOIs (10, 50, and 100) at two time periods (24 and 48 h) and included a positive control using 100 µM of H_2_O_2_. Furthermore, we performed a Western blot to assess the cleavage of caspase-3, providing support for the occurrence of apoptosis.

To maintain clarity, we have included a summary of the revised figures, which are referenced from the original manuscript, at the end of this response letter.

**Title name**

**Reviewer #3**

*Comment: Title A more factual and informative title could be: “Pathogenic Leptospira skew MoDC-CD4 T cells response towards anti-inflammatory IL-10 and Treg instead of protective IFN-g and TH1 by saprophytic strains” or substituted by the Authors’ short title “Impaired functions of human-MoDCs and induction of Treg cell by pathogenic Leptospira*

**Response**: Thank you for your suggestion.

In accordance with the reviewer’s recommendation, we have revised the title to: “Impaired Functions of Human Monocyte-Derived Dendritic Cells and Induction of Regulatory T cells by Pathogenic *Leptospira*”.

**Abstract**

**Reviewer #3**

*Comment: Abstract to rewrite to better show the novelty of the study.
The first part of the study is a confirmation of published data obtained with other serovars, showing that pathogenic strains less activate MoDCs than saprophytes. The abstract should be reformulated to put the emphasis on the novel results showing that pathogenic leptospires in contrast to saprophytic strains, skew the DC/T response to anti-inflammatory cytokines and Treg response. The sentence about apoptosis seems overstated. Please reformulate the last sentence so that the reader can understand how this study may aid the vaccine development.*

**Response:** We have meticulously restructured the abstract to accentuate the novel findings of our study in lines 22-45 of the revised manuscript.

Firstly, we have revised the background and emphasized the significance of dendritic cells in the context of leptospirosis. We have included the previously published data demonstrating that pathogenic strains activated MoDCs to a lesser extent than non-pathogenic strains. Secondly, we have rewritten the results of our experiments. We outlined the results to show novel insights into the interplay of MoDCs and T cells. Moreover, we have added the results of MoDCs apoptosis including various MOIs, different time points, and cleavage of caspase-3. Additionally, we have removed the results of the phagocytosis part from the revised manuscript as suggested by the reviewer. At the end, we have rephrased the concluding section to elucidate the significance of the findings. Specifically, we focused the benefit of the study on the new knowledge of the pathogenesis of leptospirosis and its application to vaccine development by modulating DC response.

**Author Summary**

Lines: 48-62, in response to reviewer comments, we have conducted additional experiments on MoDC apoptosis and removed the results of the phagocytosis experiment. Therefore, we have added the newly obtained results to the manuscript.

**Introduction**

**Reviewer #3**

*Comment: Line 93, the sentence “Additionally, LPS of pathogenic leptospires reduced CD40 expression and cytokine production of DCs, partially via the TLR4 and TRIF pathways” is inaccurate: in this ref only bacteria and not LPS have been used, and the LPS effect on TLR4 and TRIF is only true in murine macrophages since in human cells leptospires do not stimulate TLR4. Therefore, the sentence should be reformulated to mention the human MoDCs, which are more activated upon stimulation with the saprophytic strains compared to the pathogenic strains.*

**Response:** We appreciate your insightful comments and are grateful for the opportunity to rectify the inaccuracies in our manuscript. We have revised the introduction as follows:

Lines: 96-101, “Moreover, a pathogenic strain of leptospires demonstrated reduced activation of human MoDCs compared to a non-pathogenic strain, as indicated by the lower percentage of CD83^+^ cells. Notably, pathogenic leptospires induced a lower production of both pro- and anti-inflammatory cytokines than the non-pathogenic strain [Ref R1]. However, it is worth noting that the results were not consistent for all the pathogenic strains examined in that study.”

[Ref R1]: Cagliero J, Vernel-Pauillac F, Murray G, Adler B, Matsui M, Werts C. Pathogenic leptospires limit dendritic cell activation through avoidance of TLR4 and TRIF signaling. Front Immunol. 2022;13:911778.

*Comment: Line 100, A similar study on MoDCs activation upon infection with saprophytic and pathogenic Leptospira strains has already been published (ref missing). Here the authors should focus and study the consequences on CD4 T cell response of pathogenic Leptospira limiting DC activation, compared to the effect of saprophytic that stimulate better the MoDC-T cell response.*

Response: We sincerely appreciate your valuable feedback, which has guided us in refining the focus of our investigation to address the crucial aspects of the MoDCs-CD4^+^ T cell responses. Although a study similar to ours, focusing on MoDC activation upon infection with saprophytic and pathogenic *Leptospira* strains, has already been published [Ref R1], we have taken a slightly distinct but more rigorous approach by utilizing two different strains from both the pathogenic and non-pathogenic *Leptospira* groups. The alignment of results across various studies, showing a consistent pattern in different strains within each pathogenic and non-pathogenic group, plays a crucial role in reinforcing the validity of our derived conclusions, which are not attributed to strain variation.

In line with the reviewer's insightful suggestion, our research will now prioritize investigating the implications of limited DC activation caused by pathogenic *Leptospira* on the subsequent CD4^+^ T cell responses. In addition, we carefully compared this with the effect of saprophytic strains, which were shown to stimulate more robust CD4^+^ T cell responses. By addressing this specific mechanism, our study may contribute to a deeper understanding of the intricate interplay between *Leptospira* strains, DC activation, and subsequent T-cell responses. We have revised the introduction as follows:

Lines: 99, we have cited a pertinent reference [Ref R1].

[Ref R1] Cagliero J, Vernel-Pauillac F, Murray G, Adler B, Matsui M, Werts C. Pathogenic leptospires limit dendritic cell activation through avoidance of TLR4 and TRIF signaling. Front Immunol. 2022; 13:911778.

Lines: 103-106, we have adjusted the focus of the study towards the MoDCs-CD4^+^ T cell responses:

“Therefore, this study aimed to compare the effects of viable pathogenic (*L. interrogans* serovar Pomona and a clinical isolate) and non-pathogenic (*L. biflexa* serovar Patoc and *L. meyeri* serovar Ranarum) leptospires on human MoDCs and their influence on CD4^+^ T cells responses.”

*Comment: Lines 101-104, should be put in the abstract rather than at the end of introduction.*

**Response:** Thank you for your valuable suggestion. We have taken your feedback into consideration. Lines 101-104 have been removed from the introduction section of the original manuscript, and we have also taken the opportunity to incorporate and rephrase that content into the abstract section, ensuring that the essential aspects of our study receive prominent emphasis. In addition, we have articulated our findings in the revised introduction as follows:

Lines: 106-107, “Our results reveal a novel strategy that may be utilized by pathogenic leptospires to evade host immune responses.”

These adjustments ensure that the essential aspects of our study are highlighted in the introduction.

**Methods and results**

**Reviewer #1**

*Comment: Authors need to standardize that cells were “INFECTED” and not “STIMULATED”; both terms are being used to indicate the same thing.*

**Response**: Thank you for bringing the inconsistency in our terminology to our attention. To address this issue, we have replaced the term “stimulated and stimulation” with “infected and infection” accordingly throughout our manuscript to accurately convey the cellular interaction.

*Comment: Lines 299-312, Authors state that pathogenic strains are mostly visible and attached outside MoDCs compared to non-pathogenic strains, located “intracellularly”. I cannot see the same only by visualizing the presented images. Apparently, figure 3A indicate: PO=PA and CI=RA.*

*Comment: Fluorescence quantitative analysis of different z-stack images obtained in confocal microscopy is necessary for supporting the author’s statement.*

*Comment: Additionally, CFU phagocytosis assay, using antibiotics a few minutes after infection (to eliminate extracellular bacteria) would be helpful to clarify if bacteria are being internalized or not.*

*Reviewer #3 Comment: removing parts* *that are not essential for the purpose of this study (transcriptomic and phagocytosis). Indeed, the transcriptomic study is not properly explained nor presented. The interesting Cox2 result does not bring much to this study and could better be kept for another article, exploiting in detail the transcriptomic results. The “phagocytic” part is not properly conducted. Authors did not do gentamicin assays, so they probably only measured Leptospira-associated bacteria by cytometry. The “index” factor of phagocytosis is not appropriate to study live and motile bacteria. If intracellular Leptospira are observed by confocal microscopy (please add the non-infected MoDCs control), it does not mean phagocytosis, which relies on actin rearrangements that have not been evidenced by other groups who showed active entry of leptospires in macrophages, despite of cytochalasin treatment.*

**Response**: We greatly appreciate your thorough review and invaluable feedback on our manuscript. Your insights regarding the phagocytosis experiment in our manuscript are well taken.

After careful consideration, we concur with reviewer #3’s suggestion to exclude the phagocytosis experiment from our manuscript, as it is not essential for the objectives of this study. Specifically, we did not conduct a CFU phagocytosis assay using gentamicin to eliminate extracellular bacteria, nor did we perform fluorescence quantitative analysis using confocal microscopy to confirm bacterial internalization, both of which are crucial for assessing the occurrence of phagocytosis. We also agree that determining the intracellular location of leptospires does not conclusively indicate internalization via the phagocytosis process. As such, we acknowledge the limitations in our current experimental setup for accurately evaluating bacterial internalization via phagocytosis. Consequently, we have decided to remove the sections related to the phagocytosis experiment from the revised manuscript, including the Methods section (Lines 202-215 in the original manuscript), the results of phagocytosis (Lines 299-312 in the original manuscript), and Fig 3. We firmly believe that the remaining findings in our study continue to make a valuable contribution to the existing body of knowledge in the field.

*Comment: An apoptotic positive control is missing in these experiments, e.g. staurosporine, Viscum album, etc.*

**Response:** Thank you for your insightful comment. We appreciate your suggestion and have taken action to address it. We conducted additional apoptosis experiments using hydrogen peroxide, which has previously been used to induce apoptosis in MoDCs according to the additional reference [Ref R2] now included in the revised manuscript. Specifically, we have incorporated the results of the apoptosis experiment, employing hydrogen peroxide at a concentration of 100 µM, as a positive control in the revised manuscript.

Lines: 171-172, “Immature MoDCs treated with 100 µM hydrogen peroxide (H_2_O_2_) were used as a positive control.”

[Ref R2] Verhasselt V, Goldman M, Willems F. Oxidative stress up-regulates IL-8 and TNF-alpha synthesis by human dendritic cells. European Journal of Immunology.1998;28(11):3886-90.

*Comment: Also, analysis at different time points after infection is necessary to support the authors' statement that pathogenic strains induce apoptosis in MoDCs. By the way, the time-point after infection is not clear in the text/legend.*

*Comment: Likewise, different MOIs must be used in these experiments to help authors support their conclusion. Again, no mention of MOI used in these experiments.*

**Response:** We sincerely appreciate your thoughtful feedback. We apologize for any confusion that may have arisen from the lack of clarity in the initial text and figure legends. To strengthen the robustness of our findings, we conducted apoptosis analyses at MOIs of 10, 50, and 100, with the timepoint of 24- and 48-h post-infection. It is important to note that we used MoDCs treated with H_2_O_2_ as a positive control at each MOI and time point. We have revised the methods and results sections, including the figure legend in the revised manuscript.

Lines: 169-178, we have provided a detailed method for MoDCs apoptosis assay.

Lines: 311-355, we have presented the results of MoDCs apoptosis at different MOIs and time points in the result section and Fig 2 legend, respectively.

Lines: 524-544, we also have presented the discussion of MoDCs apoptosis.

*Comment:* *The letters system adopted by the authors to indicate statistical difference is not obvious.*
**Response**: We appreciate your observation concerning the clarity of the symbol system used to indicate statistical differences. In response, we have revised all figures and implemented a clearer and more easily understandable symbol system to represent statistical significance in the legends of Figures 1-5 in the revised manuscript. In general, statistical significance is now indicated as follows: * *P* < 0.05; ** *P* < 0.01; *** *P* < 0.001; **** *P* < 0.0001; # *P* < 0.05 compared to the negative control, such as uninfected MoDCs, depending on the experimental design.

*Comment: Authors named conditions in the text as MoDCs-P and MoDCs-NP. However, the figures do not follow the same nomenclature. Standardizing the condition’s names on both text and figures would help for faster comprehension.*

**Response**: We have taken your observation into consideration and made necessary adjustments. To ensure consistent and coherent presentation, we now refer to MoDCs infected with pathogenic leptospires as “MoDCs-P” and MoDCs infected with non-pathogenic leptospires as “MoDCs-NP” throughout both the text and the corresponding figures. This alignment will facilitate faster comprehension and maintain consistency between the text and figures.

*Comment: Lines 333-342, I'm wondering why the authors didn't present figures S4-S5 together in Fig4. Cell death analysis needs different approaches/analyses to be undoubted. However, there are some concerns, regarding cell death, that need to be further explored by the authors. Both P and NP strains induced a very low rate of apoptosis. Some additional controls and conditions are required for authors to highlight the biological relevance of apoptosis induced by leptospire strains.*

**Response:** Thank you for your thoughtful comments regarding the presentation of our figures and the analysis of cell death in our manuscript. In response to your query, allow us to elucidate our rationale for the arrangement of figures and our approach to cell death analyses.

Initially, we focused on evaluating the potential of leptospires to induce cell death at an MOI of 100, 24 h post-infection. The results demonstrated that in contrast to non-pathogenic leptospires, pathogenic strains induced cell death, as determined by the MTS assay. Therefore, we have maintained the percentage of viable MoDCs infected with leptospires, as assessed by MTS assay, in supplement 4 Fig in the revised manuscript. Subsequently, our focus shifted towards understanding the effect of leptospires on mature MoDCs in terms of their capacity to induce apoptosis. In the revised manuscript, we have relocated the scatter plots, which serve as a visual representation of the MoDC apoptosis induced by the various strains of leptospires at an MOI of 100 [formerly in S5 of the original manuscript], into the main figure (now Fig 2A).

In response to your concerns and to ensure a more comprehensive investigation of apoptosis, we extended our analysis. Specifically, we examined apoptosis at two distinct time points (24 and 48 h) and evaluated it across three different MOIs (10, 50, and 100). Moreover, we incorporated a positive control using 100 µM hydrogen peroxide (H_2_O_2_) to enhance the robustness of our findings. To enhance the clarity of our presentation, we have already integrated the scatter plots depicting apoptosis (formerly in S5 of the original manuscript) into the main text as Fig 2A in the revised manuscript. Moreover, we have demonstrated a significantly higher percentage of apoptosis of MoDCs-P compared to MoDCs-NP in an MOI- and time-dependent manner, as determined by flow cytometry, in Fig 2B of the revised manuscript. Notably, the highest percentage of apoptosis was observed, reaching approximately 80%, when MoDCs were infected with pathogenic *Leptospira* serovar Pomona at an MOI of 100 for 48 hours, as depicted in Fig 2B in the revised manuscript.

*Comment: It is not clear for how long MoDCs were prior infected before co-culture with CD4+ T cells. Figure S7 should be placed within Fig6 for a more accurate illustration of the events taking place*.

**Response:** We apologize for any confusion caused by the lack of explicit information in the manuscript. To address this concern, we have revised the manuscript to provide clearer details. The MoDCs were infected with pathogenic and non-pathogenic leptospires for a period of 24 h. Following this 24-h infection, the infected MoDCs were co-cultured with CD4^+^ T cells.

Regarding the placement of Fig S7 in the original manuscript, we agree with your suggestion to incorporate it within Fig 4A in the revised manuscript for a more accurate illustration of the events taking place. In the revised manuscript, we have included Fig S7 in the original manuscript as part of Fig 4A, thereby providing a more cohesive and comprehensive representation of the experimental findings.

Lines: 214-215, we have also elaborated on the duration of MoDCs infection before co-culture with CD4^+^ T cells in the Methods section as follows:

“After 24-h infection of MoDCs with leptospires, infected MoDCs were collected and washed three time with PBS with 1% penicillin and streptomycin.”

*Comment: Lines 400-414, In the results section, the authors highlighted that PTGS2 was the most intriguing DEG but didn’t explain Why. Although PTGS2’s role is further explored in the discussion, it would be important for readers a brief paragraph or sentence explaining why authors end up pondering PTGS2's importance in the current study. Also, Fig7A is poorly explored. It does not inform much other than that authors have chosen the PTGS2 (again, for a non-mentioned reason).*

**Response:** Thank you for your thoughtful comment regarding the clarity and explanation of our results related to the *PTGS2* gene. We appreciate your feedback, and we acknowledge the need to provide a clear rationale for our focus on the *PTGS2* gene and its significance in our study.

In the results section, *PTGS2* or *COX-2* gene was one of the highly upregulated genes in the differentially expressed gene (DEG) because the role of the *PTGS2* gene in influencing T cell responses is notably consistent with our findings in this study. Specifically, *PTGS2* gene expression in MoDCs-P likely contributes to the generation of prostaglandins, including PGE2. These prostaglandins can have immunosuppressive effects in terms of the reduction of T cell proliferation and the promotion and expansion of Tregs [Ref R3]. These mechanisms align with our findings, as we observed decreased T cell proliferation (Fig 4B in the revised manuscript) and induction of Tregs (Fig 3 in the revised manuscript] in response to *PTGS2*-expressing MoDCs-P. Moreover, the significant upregulation of the *PTGS2* gene was observed in the immune cells of non-survival hamsters infected with pathogenic leptospires [Ref R4]. These explanations should help readers to understand the context behind our emphasis on this *PTGS2* gene.

Lines: 412-416, we have incorporated the explanation to enhance the understanding of our emphasis on the *PTGS2* gene in the result section as follows:

“The gene encoding prostaglandin-endoperoxide synthase 2 (*PTGS2*), also known as cyclooxygenase-2 (COX-2), emerged as a compelling target for further investigation because its PGE2 product has been previously shown to reduce T cell proliferation and promote the activation of Tregs [Ref R3], observations that are consistent with our findings in MoDCs-P.”

[Ref R3] Sreeramkumar V, Fresno M, Cuesta N. Prostaglandin E2 and T cells: friends or foes? Immunol Cell Biol. 2012;90(6):579-86.

[Ref R4] Vernel-Pauillac F, Goarant C. Differential cytokine gene expression according to outcome in a hamster model of leptospirosis. PLoS Negl Trop Dis. 2010;4(1):582.

Additionally, we appreciate your observation regarding Fig 7A in the original manuscript and its limited exploration. To enhance the clarity and depth of our presentation, we have opted to remove Fig 7A from the revised manuscript. Instead, we have incorporated a table (Table 1 in the revised manuscript) that highlights the top 5 upregulated and downregulated genes in MoDCs-P. This table offers a more focused and informative representation of the differential gene expression.

**Reviewer #2**

*Comment: Line141, on day7, the immature DCs were stimulated with leptospires (100 MOI). When this DC maturation occurs in vitro? How is their maturation determined?*

**Response:** We truly appreciate your thoughtful feedback. After stimulating the immature MoDCs with leptospires at an MOI of 100, MoDC maturation was observed in vitro at 24 h post-infection.

The maturation of MoDCs can be determined by evaluating specific phenotypic and functional changes that occur upon stimulation (as shown in Figure 1 in the revised manuscript) as follows:

1. Surface marker expression: CD83 is one of the most prominent surface markers for fully matured dendritic cells. CD80 and CD86 are co-stimulatory molecules, which are involved in antigen presentation and T cell activation. When MoDCs reach a mature state, they exhibit increased expression of these molecules. In our study, we assessed CD83, CD80, and CD86 to evaluate MoDC maturation.
2. Cytokine secretion: Mature MoDCs produce and secrete various cytokines involved in immune activation and polarization. We measured the secretion of IL-6, IL-10, and IL-12p70.
3. Antigen presentation capability: Mature MoDCs have an enhance capacity to present antigens to T cells, as indicated by increased expression of MHC class II molecules. In our study, we assessed the expression of HLA-DR on MoDCs.
4. Morphological changes: Mature MoDCs typically display a rough surface with multiple pseudopodia, whereas immature MoDCs have a round and smooth surface. Although we observed morphological changes upon infection with leptospires, these changes could not be accurately quantified. Therefore, we did not include this characteristic of mature MoDCs in our study.

*Comment: For the whole study, author needs to provide detailed method of generation of moDC in vitro and how the replated in 24 well plate for leptospiral stimulation. What cellular detachment technique were used during this process? Did the author stimulate the cells with Leptospira in complete medium with antibiotics? Details of the methods will be helpful to evaluate the observed phenomena in more details.*

**Response:** Thank you for your valuable comment and your interest in the details of our experimental methods. We would like to provide a more detailed description of the methods used for the generation of MoDCs in the Methods section of the revised manuscript as follows:

Lines: 144-152, “On day 7, MoDCs were detached from flasks by gently pipetting using cold phosphate buffered saline (PBS) and tapping the flasks firmly. Subsequently, the immature MoDCs were centrifuged at 500 ×g for 10 min and resuspended in the RPMI 1640 medium without antibiotics. Prior to seeding in the plates, CD1a and CD83 markers were assessed to ensure that cells remained as immature MoDCs. Then, MoDCs (at a density of 106 cells/ml) were seeded in 24-well plates (Corning^®^). Following that, the immature MoDCs were infected with leptospires at multiplicities of infection (MOIs) of 10, 50, and 100 in RPMI 1640 culture medium without antibiotics. Next, the plates were centrifuged at 500 ×g for 10 min to synchronize of infection [Ref R5]

[Ref R5] Yamaguchi T, Higa N, Okura N, Matsumoto A, Hermawan I, Yamashiro T, et al. Characterizing interactions of *Leptospira interrogans* with proximal renal tubule epithelial cells. Bmc Microbiol. 2018;18.

*Comment: Line120, why it needs an aerobic condition to grow? If so, how it was grown in culture in aerobic condition?*

**Response:** We greatly appreciate your inquiry regarding the growth conditions of leptospires and would like to provide a clear explanation. *Leptospira* is classified as an obligate aerobic spirochete. In alignment with their optimal growth conditions, leptospires were cultured in liquid Ellinghausen-McCullough-Johnson-Harris (EMJH) medium (BD Difco^TM^) supplemented with 10% bovine serum albumin solution at 30°C under aerobic conditions without agitation until the exponential growth phase was reached (~ 2×10^8^ cells/ml) [Ref R6] (lines 120-123).

[Ref R6] Zuerner RL. Laboratory maintenance of pathogenic *Leptospira*. Curr Protoc Microbiol. 2005; Chapter 12: Unit 12E1.

*Comment: Line206-207, Cells incubated at 4 degree C used as a negative control. Please provide detail of doing so. For phagocytosis assay, CFSE stained bacteria were used. How could the author determine the bacterial multiplication with CFSE?*

**Response:** We greatly appreciate your inquiry regarding specific aspects of our study. In line with your query, cells incubated at 4 degrees Celsius were employed as a negative control for the phagocytosis assay. The rationale behind this approach is that MoDCs exhibit diminished phagocytic activity at lower temperatures. Therefore, we used cells incubated at this condition as a negative control for phagocytosis. This measure was implemented to minimize the potential for cell-mediated phagocytosis of the negative control. In terms of determining bacterial multiplication using CFSE, we initially relied on the phagocytosis index as outlined in reference R7.

However, our methodology did not involve the use of gentamicin to eliminate extracellular bacteria, a point thoughtfully raised by Reviewers #1 and #3. After careful consideration, we agree with the suggestion of reviewer #3 to omit the phagocytosis experiment from the manuscript, as it does not serve as an essential component for the objectives of this study. Consequently, we have removed the content pertaining to the phagocytosis assay from the manuscript. Specifically, we have removed the phagocytic activity of MoDCs in the method section (lines 202-215 of the original manuscript) and the phagocytic ability of immature MoDCs in the result section (lines 299-313 of the original manuscript). Additionally, we have removed Fig 3 and Supplement Fig 3 from the original manuscript.

[Ref R7] Mason LMK, Koetsveld J, Trentelman JJA, Kaptein TM, Hoornstra D, Wagemakers A, et al., *Borrelia miyamotoi* activated human dendritic cells and elicits T cell responses. J Immunol. 2020;204(2):386-93.

**Reviewer #3:**

*Comment:* *adding isotype controls. Indeed, FMO and isotypes were only included when appropriate (line 222) but results are not shown here. Yet, threshold of fluorescence should be done using isotypes and not unstained cells. This should be performed and, at least, shown in sup data. The analysis should be raw MFI instead of “relative MFI”. It would permit to visualize the basal expression of the different activation markers in non-infected cells. Also, why did the authors not study CD40 up-regulation?*

**Response:** We appreciate your concern about the inclusion of isotype controls and the importance of establishing florescence intensity thresholds. We acknowledge the value of visualizing basal expression levels of activation markers in non-infected cells. To address this, we have included raw MFI, fluorescence minus one (FMO), and isotype control data in supplementary Figs 2 and 3.

While we agree that using MFI is a standard analysis, we chose to employ relative MFI in our analysis for specific reasons. Relative MFI, as implemented following Ref R8, offers the advantage of normalizing the fluorescence intensity values to a negative control (uninfected MoDC groups). This normalization facilitates more accurate comparisons between various samples that were collected and analyzed on different rounds of assay. Additionally, genetic variation among human donors results in diverse MoDC responses to leptospires. Therefore, this normalization accounts for technical variations and enables more reliable comparisons. Furthermore, relative MFI can effectively demonstrate increases or decreases in the expression of surface markers, even in the presence of variation in basal levels.

Our study primarily focused on the impact of leptospires on MoDC maturation and T-cell activation. We assessed CD83, a prominent marker for mature MoDCs, along with CD80 and CD86, which serve as representative co-stimulatory molecules on mature MoDCs. CD80 and CD86 act as critical co-stimulatory molecules, directly interacting with CD28 and CTLA-4, thereby influencing the strength and nature of T cell activation, proliferation, and effector function. We did not evaluate the expression of CD40, as the detection of CD80 and CD86 as well as CD83 and HLA-DR on MoDCs (as shown in Fig 1A of the revised manuscript) was deemed sufficient to achieve our objective of determining the differential effects of pathogenic and non-pathogenic leptospires on MoDC maturation and their subsequent impact on T cell responses.

[Ref R8] Lopez-Relano J, Martin-Adrados B, Real-Arevalo I, Lozano-Bartolome J, Abos B, Sanchez-Ramon S, et al. Monocyte-Derived Dendritic Cells Differentiated in the Presence of Lenalidomide Display a Semi-Mature Phenotype, Enhanced Phagocytic Capacity, and Th1 Polarization Capability. Front Immunol. 2018; 9:1328.

*Comment*: *removing parts that are not essential for the purpose of this study (transcriptomic and phagocytosis). Indeed, the transcriptomic study is not properly explained nor presented. The interesting COX-2 result does not bring much to this study and could better be kept for another article, exploiting in detail the transcriptomic results. The “phagocytic” part is not properly conducted. Authors did not do gentamicin assays, so they probably only measured Leptospira-associated bacteria by cytometry. The “index” factor of phagocytosis is not appropriate to study live and motile bacteria. If intracellular Leptospira are observed by confocal microscopy (please add the non-infected MoDCs control), it does not mean phagocytosis, which relies on actin rearrangements that have not been evidenced by other groups who showed active entry of leptospires in macrophages, despite of cytochalasin treatment.*
**Response:** We greatly appreciate your valuable feedback on our manuscript. Your insights regarding the phagocytosis experiment are well taken.

After careful consideration, we agree with you to remove the phagocytosis experiment from the manuscript, as it is not essential for the core objectives of this study.

However, we believe it is important to include the transcriptomic results to demonstrate the differential gene expression in MoDCs infected with pathogenic and non-pathogenic strains. Notably, the *PTGS2* (or *COX-2*) gene was the most intriguing differentially expressed gene (DEG). The role of the *PTGS2* (or *COX-2*) gene in modulating T cell responses aligns with our findings in this study. Specifically, the up-regulated expression of the *PTGS2* gene in MoDCs infected with pathogenic leptospires (MoDCs-P) likely contributes to the generation of prostaglandins, including PGE2. These prostaglandins can exert immunosuppressive effects, such as inhibiting T cell proliferation and promoting the activation and expansion of Tregs (Fig 3 in the revised manuscript) [Ref R9]. These mechanisms corroborate our observations of reduced T cell proliferation (Fig 4B in the revised manuscript) and the induction of Tregs (Fig 3 in the revised manuscript) in response to *PTGS2*-expressing MoDCs-P. Moreover, the levels of PGE2 in the sera of patients with acute leptospirosis were higher than those of healthy donors (Fig 6 in the revised manuscript), suggesting that pathogenic strains might induce the PTGS2/PGE2 pathway.

Lines: 412-416, we have incorporated the explanation to enhance the understanding of our emphasis on the PTGS2 gene in the result section.

Lines: 508-523, we have discussed the potential role of the PTGS2/PGE2 pathway that may be relevant to our findings in the discussion section.

[Ref R9] Sreeramkumar V, Fresno M, Cuesta N. Prostaglandin E2 and T cells: friends or foes? Immunol Cell Biol. 2012;90(6):579-86.

*Comment: The first part of the study is a confirmation of published data obtained with other serovars, showing that pathogenic strains less activate MoDCs compared to saprophytic. Please rephrase carefully: MoDCs exposed to pathogenic leptospires show less activation than those exposed to saprophytic leptospires. The cells are still activated by pathogenic strains, they are just less so, as has been shown in the literature. Figure 1 and 2 could be presented in the same figure.*

**Response:** We greatly appreciate your invaluable suggestions. In The first part of our study, our results corroborate the previously published findings obtained from distinct serovars. These consistent findings indicated that MoDCs infected with pathogenic strains exhibit less activation compared to those infected with non-pathogenic strains. To clarify, we have used the term “partial impairment” in describing MoDCs-P maturation to indicate that these cells are still activated by pathogenic strains, albeit to a lesser extent than MoDCs-NP.

Lines: 290-292, we have rewritten as follows:

“These findings suggest that pathogenic leptospires can activate MoDC maturation, albeit to a lesser extent than non-pathogenic strains, potentially leading to partial impairment of MoDCs-P maturation.”

Additionally, we have taken the step of merging Fig 1 and Fig 2 into a single combined Fig 1 in the revised manuscript. This adjustment aims to enhance the overall presentation and clarity of our study.

*Comment: confirming the MoDC apoptosis phenotype with additional experiments. Please add a positive control (**staurosporine?) to be able to gate the apoptotic cells. Moreover, only a maximum of 10% of MoDCs infected with pathogenic strains undergo early apoptosis, and almost none are detected in late apoptosis: it would then be interesting to measure apoptosis with other methods (caspase 3 cleavage by WB, or Caspase 3/7 activity kit) to really assess apoptosis of MoDCs upon infection with pathogenic strains. A kinetic would be required too. Then the meaning of this phenotype regarding the MoDCs functions should be carefully discussed, avoiding overstatements and references not always appropriate (471-477).*

**Response:** We greatly appreciate your thoughtful suggestions aimed at further validating the MoDC apoptosis phenotype and enhancing the robustness of our findings. To provide a positive control, we have incorporated the use of 100 µM H_2_O_2_. Hydrogen peroxide has previously been used to induce apoptosis in MoDCs [Ref R10]. The results of the positive control were useful for gating apoptotic cells using Annexin V and 7-AAD staining in flow cytometry.

Additionally, in order to provide more convincing data regarding the induction of apoptosis by leptospires, we conducted a discrete kinetic analysis by measuring apoptosis of MoDCs infected with leptospires at MOIs of 10, 50, and 100 for 24 and 48 hours. Our findings demonstrated that pathogenic strains induced significantly higher levels of MoDC apoptosis than non-pathogenic leptospires in a dose- and time-dependent manner. Notably, the highest percentage of early apoptosis reached approximately 80% when MoDCs were infected with pathogenic *Leptospira* serovar Pomona at an MOI of 100 for 48 hours, as depicted in Fig 2B in the revised manuscript.

We acknowledge the importance of incorporating alternative methods to further confirm the induction of MoDC apoptosis upon infection with leptospires. Therefore, we performed Western blot analysis to detect the caspase-3 cleavage in MoDCs infected with leptospires at an MOI of 100 for 48 h. The results revealed higher levels of cleaved caspase-3 in MoDCs infected with pathogenic leptospires compared to non-pathogenic strains, as illustrated in Fig 2C in the revised manuscript. This observation aligns with the results obtained via flow cytometry.

Lines: 169-193, we have revised the relevant sections of the methods pertaining to the apoptosis assays.

Lines: 311-355, we have revised the relevant sections of the results pertaining to the apoptosis assays. The figures of MoDC apoptosis have been removed from Fig 3A and B in the original manuscript and are now included in Fig 2A and B in the revised manuscript.

Lines: 524-544, we have revised the discussion pertaining to the apoptosis assays.

[Ref R10] Verhasselt V, Goldman M, Willems F. Oxidative stress up-regulates IL-8 and TNF-alpha synthesis by human dendritic cells. European Journal of Immunology.1998;28(11):3886-90.

*Comment: Line 259, the title of paragraph is not accurate. It should be “Pathogenic leptospires less activate MO-DC maturation than saprophytic leptospires. Fig 1: why did you use “relative MFI”? You would beneficiate from showing MFI, and please add isotype controls or FMO.*

**Response:** We really appreciate your thorough review of the manuscript. The title of paragraph has been revised as follows:

Lines: 282-283, “Pathogenic leptospires less activate MoDC maturation than non-pathogenic leptospires”

As mentioned previously, the use of relative MFI allows for the normalization of MFI values to a negative control (unstimulated MoDC groups). This normalization facilitates more precise comparisons among different samples. Additionally, due to genetic variation among human donors, MoDC may respond differently to leptospires. Therefore, this normalization decreased technical variations and enhances the reliability of comparisons among different batches of samples performed in an inter-assay manner. Relative MFI effectively highlights both increases and decreases in the expression of surface markers. Moreover, we have included FMO controls for MoDC maturation and isotype controls for intracellular cytokines in CD4^+^ T cells in the supplementary materials (S2 and S3 Figs in the supplement of the revised manuscript).

*Comment: Line 299, no gentamicin elimination of leptospires for the phagocytic assay. Therefore, better speak about Leptospira associated bacteria and internalization.*

*Comment: Line 333, this is not “phagocytosis” observed in cytometry but Leptospira associated with cells*

**Response:** We have removed the phagocytosis assay from the manuscript, as suggested by the reviewer #3.

*Comment: Line 385, the conclusion seems overstated. There is no demonstration of “inhibition”. Data showed that MoDCs stimulated with saprophytic strains induce T cell proliferation in contrast to MoDCs infected with pathogens.*

**Response:** We appreciate your attention to detail and the valuable feedback you have provided. We apologize for any potential overstatement in our conclusion. Our experiments revealed that CD4^+^ T cells exhibited only slight proliferation when activated by MoDCs-P, in contrast to MoDCs-NP (Fig 4B in the revised manuscript). Consequently, in both our results and conclusion, we refrained from using the term “inhibition” as it could be considered an overstatement. Instead, we consistently employed the phrase “fail” to induce CD4^+^ T cell proliferation” (lines 31, 357, 390, 476, 564) in the revised manuscript.

*Comment: Line 403: after 24h of stimulation?*

**Response:** We greatly appreciate your thorough review of the manuscript. In the RNA sequencing analysis, MoDCs were infected with pathogenic and non-pathogenic leptospires for 4 hours. We selected this specific time point to study the genes of MoDCs involved in early immune responses to leptospires.

Lines: 361-362, we have written as follows:

“The T-cell activation-induced markers and cytokines were measured by flow cytometry after 48 h co-culture.”

**Discussion**

**Reviewer #1**

*Comment: Still regarding PTGS2, authors say in the discussion (lines 521-524) that their data corroborates to literature that PTGS2 is regulated by NF-kB activation. However, the authors do not present any data regarding NF-kB activation, instead, they mention some unshown transcriptome data which would suggest NF-kB downregulation. Wouldn’t NF-kB downregulation afterward result in lower levels of PTGS2? That is why NF-kB activation and additional pathways must be better explored (writing/experimentally) by the authors before they propose such a sequence of events after infection with P or NP strains of leptospires, illustrated in figure9.*

**Response:** In response to your comment regarding NF-kB activation, we apologize for any confusion caused by our statement in the discussion section. Based on transcriptomic data derived from MoDCs infected with leptospires at a single time point, we were unable to establish a direct association between the up-regulation of the *PTGS2* (*COX-2*) gene and the NF-kB pathway. We acknowledge the need for further investigation of NF-kB activation and other potential pathways to enhance our comprehension of the sequence of events following infection with pathogenic or non-pathogenic leptospires. Consequently, we have removed the content pertaining to the NF-kB pathway from both the discussion and Fig 9 in the original manuscript. We appreciate your suggestion and plan to investigate the activation of *PTGS2* (*COX-2*)/PGE2 in our future studies, with the aim of achieving a more comprehensive understanding of the underlying mechanisms.

**Reviewer #2:**

*Comment: This study utilized monocyte derived dendritic cells to describe the leptospiral pathogenesis. It would be great if observed phenomena can be correlated through an in vivo study.*

**Response:** We appreciate your constructive input. This study employed human MoDCs to provide valuable insights into the pathogenesis of leptospirosis in humans. We recognize the limitation of this study, which relies on MoDCs in a controlled *in vitro* setting and cannot fully replicate the complexity of *in vivo* systems. While we acknowledge the importance of validating these findings in an *in vivo* study, ethical constraints preclude the direct use of human subjects for infection experiments aimed at determining the effect of leptospires on human DCs and the interaction between mature DCs with CD4^+^ T cells. In line with this suggestion, further studies to investigate dendritic cell responses to pathogenic leptospires should be conducted using well-established susceptible animal models for leptospirosis, such as TLR4 deficient C3H/HeJ mice or TLR4 knockout mice. These models will allow us to explore the interaction between pathogenic leptospires and dendritic cells in a more physiologically relevant *in vivo* environment. However, it is important to note that while animal models provide valuable insights, the results obtained from these models may not always perfectly mirror the responses seen in humans.

*Comment: Line102-104, authors are requested to provide justification why both interferon gamma production and increase of CD4+ cells were induced by the nonpathogenic strain. Does nonpathogenic Leptospira sp. Infection require induction of an adaptive immunity, or it is efficiently cleared by the innate immune cells?*

**Response:** We appreciate your valuable feedback. The objectives of this study aimed to determine the major role of human MoDCs and their link to the adaptive immunity. This study assessed the effect of MoDCs infected with non-pathogenic *Leptospira* in comparison to those infected with pathogenic strains on CD4^+^ T cells, a vital component of adaptive immunity. Our study revealed that infection of non-pathogenic strains in MoDCs resulted in robust proliferation of CD4^+^ T cells and the production of IFN-γ by CD4^+^ T cells, indicating the involvement of Th1 cells. Regarding the innate immunity, we have revised the discussion as follows:

Lines: 547-561, “In the context of natural human infections, there is no current evidence whether non-pathogenic strains are cleared by innate immune responses or whether the induction of adaptive immunity is required. Although saprophytic leptospires are sensitive to the alternative pathway of complement-mediated killing *in vitro* [Ref R11], it remains unclear whether this mechanism is sufficient to eradicate non-pathogenic leptospires *in vivo*. A recent study demonstrated that live saprophytic leptospires could exit human macrophage without causing cell lysis and were resistant to classical macrophage microbicidal mechanisms such as phagocytosis, autophagy, toll-like receptor-mediated reactive oxygen species (ROS), and reactive nitrogen species (RNS) production [Ref R12]. It is possible that, after exiting the macrophages, other immune strategies including Th1 response may contribute to bacterial clearance. A combination of innate and adaptive immune mechanisms might be required to eliminate non-pathogenic leptospires from the host. Therefore, our findings suggest, but do not definitely establish, that the induction of a CD4^+^ T cell response may be involved in the clearance of non-pathogenic leptospires. Additional studies are needed to elucidate the mechanisms responsible for their elimination.”

[Ref R11] Meri T, Murgia R, Stefanel P, Meri S, Cinco M. Regulation of complement activation at the C3-level by serum resistant leptospires. Microb Pathog. 2005;39(4):139-47.

[Ref R12] Santecchia I, Bonhomme D, Papadopoulos S, Escoll P, Giraud-Gatineau A, Moya-Nilges M, et al. Alive Pathogenic and Saprophytic Leptospires Enter and Exit Human and Mouse Macrophages with No Intracellular Replication. Front Cell Infect Microbiol. 2022;12:936931.

*Comment: The IL10 production was less with pathogenic strain whereas the intracellular cytokine staining of CD4+ cells showed opposite phenomenon. The authors are requested to justify the observed fact which is contradictory. Author could check the cytokines in the patient sera for all the cytokines and correlate the observed findings if it matches with the moDC/CD4+cells data.*

**Response:** We greatly appreciate your valuable feedback. To address the discrepancy between IL-10 production and intracellular cytokine staining in our study, it is essential to consider the experimental design and the cellular sources of IL-10 in each assay.

In the first experiment, we examined IL-10 production by MoDCs infected with leptospires for 24 h, representing the early stages of interaction between MoDCs and leptospires. In this context, MoDCs are the primary source of IL-10 in this experiment. In the second experiment, we investigated the intracellular cytokine levels in CD4^+^ T cells after co-culturing with MoDCs infected with pathogenic leptospires for 48 h. Importantly, IL-10 can be produced by various immune cells, including MoDCs and T cells. To definitely establish the cellular origin of IL-10 in the second experiment, we performed intracellular cytokine staining, which conclusively demonstrated that IL-10 was indeed derived from CD4^+^ T cells in the co-culture setting. Given that IL-10 in each experiment originated from distinct cell types, the results from these two experiments are not contradictory but rather provide complementary information on the cellular sources of IL-10 in the context of leptospiral infection.

We acknowledge your suggestions to validate and correlate our findings in clinical samples. Evaluating cytokine levels in the sera of patients with leptospirosis and in relevant *in vivo* models is indeed a valuable avenue for further investigation. Previous studies have reported association between serum levels of pro-inflammatory markers, such as IL-6 and TNF-alpha, and severe human leptospirosis [Ref R13]. However, it is important to emphasize that several cytokines can be produced by overlapping immune cell populations. Therefore, the detection of each cytokine in patient sera should be carefully interpreted with respect to their sources and their effects on distinct immune cells.

We chose not to include the analysis of cytokine levels in patient sera in our current study because it would be challenging to definitively establish whether these cytokines are produced by dendritic cells and their direct impact on CD4^+^ T cells. Therefore, it might be difficult to directly correlate them with our findings.

[Ref R13] Cagliero J, Villanueva S, Matsui M. Leptospirosis Pathophysiology: Into the Storm of Cytokines. Front Cell Infect Microbiol. 2018; 8:204.

*Comment: Line508-510: The expression levels of CD83 were reduced a little than that of the nonpathogens whereas the CD86 expression remain similar for all the infected groups. Based on that it is hard to conclude such process without in detail investigation.*

**Response:** We appreciate your insightful comment regarding the expression of mature MoDC markers after leptospiral infection. While the levels of CD83 and CD80 expression on MoDCs-P were slightly lower than those on MoDCs-NP, the difference was still statistically significant. However, CD86 did not exhibit a significant difference between MoDCs-P and MoDCs-NP. Based on these findings, it can be concluded that pathogenic leptospires could activate MoDC maturation but to a lesser extent than non-pathogenic leptospires. Therefore, we have revised the description of the effect of pathogenic leptospires from “impairing MoDC maturation” to “partially impairing MoDC maturation.” The possible roles of lower expression of CD83 and CD80 in T cell responses are described in the discussion section as follows:

Lines: 480-484, we have written the discussion of CD83

“Beyond serving as a prominent marker for mature DCs, CD83 also play a role in regulating T cell responses and maintaining immune system balance [Ref R14]. Previous research has shown that downregulated CD83 expression on human DCs resulted in less potent induction of T cell proliferation and reduced IFN-γ secretion by T cells [Ref R15].”

[Ref R14] Grosche L, Knippertz I, Konig C, Royzman D, Wild AB, Zinser E, et al. The CD83 Molecule - An Important Immune Checkpoint. Front Immunol. 2020;11:721.

[Ref R15] Aerts-Toegaert C, Heirman C, Tuyaerts S, Corthals J, Aerts JL, Bonehill A, et al. CD83 expression on dendritic cells and T cells: correlation with effective immune responses. Eur J Immunol. 2007;37(3):686-95.

Lines: 485-498, we have written the discussion of CD80 and CD86

“Furthermore, strong co-stimulatory signals from CD80 and CD86 are essential for the activation of naïve T cells in vitro [Ref R16]. Although CD80 and CD86 share the same receptors (CD28 or CTLA-4) on T cells, they exhibit distinct binding affinities and kinetics, leading to distinct functional outcomes. Our results demonstrated that MoDCs-P exhibited lower CD80 expression compared to MoDCs-NP, but CD86 expression levels were not significantly different. This suggests that the interaction involving CD80 may potentially affect the binding force between CD28 or CTLA-4 receptors on CD4^+^ T cells. These findings align with previous studies that showed inhibiting CD80 on mouse dendritic cells reduced the interaction forces between DCs and T cells, consequently impairing T cell activation and reducing IFN-γ secretion [Ref R16]. Given that our study focused on a single time point (24 h post-infection), it is worth noting that the abundance and differential expression of MoDC surface markers may dynamically change upon exposure to inflammatory stimuli, including leptospiral infection. Additional research is needed to elucidate the roles and mechanisms of CD80 and CD83 in MoDC responses to pathogenic leptospires.”

[Ref R16] Lim TS, Goh JK, Mortellaro A, Lim CT, Hammerling GJ, Ricciardi-Castagnoli P. CD80 and CD86 differentially regulate mechanical interactions of T-cells with antigen-presenting dendritic cells and B-cells. PLoS One. 2012;7(9):45185.

*Comment: The authors did not check the other cell death pathway. Maybe other cell death processes are important during leptospiral or any infection. Authors could cite the recent articles published (PMID: 35392072, 33221042) in line with this process and add a discussion on how cell death influences the inflammation during infections.*

**Response:** We appreciate your suggestion regarding the exploration of different mechanisms of cell death that may trigger immune responses and inflammation during infection. Our primary objectives were to investigate how MoDCs infected with pathogenic and non-pathogenic strains respond to CD4^+^ T cells. However, further investigation is needed to determine whether other types of cell death in MoDCs are induced by pathogenic leptospires. Therefore, we have revised the discuss as follows:

Lines: 537-544, we have mentioned other mechanisms of cell death induced by pathogenic leptospires in the discussion section as follows:

“However, previous research regarding other types of cell death induced by pathogenic leptospires has not been reported in human DCs. Necroptosis has been shown to be involved in the innate immune response and contribute to persistent inflammation during acute leptospirosis in C3H/HeJ mice [Ref R17]. A previous study showed that pathogenic leptospires did not induce pyroptosis in murine, bovine, human, and hamster macrophages [Ref R18]. Given that our study was limited to apoptosis mediated by caspase-3 activation and did not explore other mechanisms of cell death, further studies are warranted to provide comprehensive understanding.”

[Ref R17] Kundu S, Shetty A, Gomes-Solecki M. Necroptosis Contributes to Persistent Inflammation During Acute Leptospirosis. Front Immunol. 2022; 13:810834.

[Ref R18] Bonhomme D, Hernandez-Trejo V, Papadopoulos S, Pigache R, Fanton d'Andon M, Outlioua A, et al. *Leptospira interrogans* Prevents Macrophage Cell Death and Pyroptotic IL-1beta Release through Its Atypical Lipopolysaccharide. J Immunol. 2023;210(4):459-74.

**Reviewer #3:**

*Comment:editing/rewriting the manuscript and title to better highlight the novelty of this work. It would improve its readability, while avoiding overstatements (see minor comments). Moreover, in the manuscript (and especially in the discussion part), a lot of references are poorly corresponding to the text. The authors should carefully check each sentence and associated reference to avoid approximative citations.*

**Response:** We appreciate your invaluable suggestion. We have thoroughly reviewed and revised the manuscript as well as verified the references to ensure they align accurately with the content throughout the revised manuscript.

*Comment: Please rewrite carefully the discussion., which is not very clear and often confusing. Please avoid discussing data not shown (line 521). Please insist on the anti-inflammatory and Treg aspect.*

**Response:** We apologize for any confusion that may have arisen. We have carefully revised the discussion section to enhance clarity and accuracy, specifically by refraining from discussing data not presented between lines 521-526 in the original manuscript. We were unable to establish a direct relationship between the up-regulation of the *PTGS2* (*COX-2*) gene and the NF-kB pathway; therefore, we removed it from the revised manuscript. The result from intracellular cytokine straining of CD4^+^ T cells revealed that MoDCs-P induced IL-10 secretion and, notably, an increase in CD25^+^FoxP3^+^ Tregs. IL-10 production was also associated with Treg cell responses. IL-10 is known to act on Treg cells by maintaining the transcription factor FoxP3 and may even limit the differentiation of CD4^+^ T cells.

*Comment: Line 404, should be written “pathogenic strains induced “impaired” MoDC responses” instead of “does not induce…”*

**Response:** We appreciate your thorough review of the manuscript.

Lines: 405, we have written as follows:

“We found that pathogenic strains partially impaired MoDC responses.”

*Comment: Line 478, should be written “by lower” instead of “by low”*

**Response:** Thank you for catching that error.

Lines: 477-478, we have written as follows:

“MoDCs-P exhibited lower expression levels of CD83 and CD80 compared to MoDCs-NP.”

*Comment: Line 481-492, The paragraph about the discrepancy is confusing. Virulence and pathogenicity should not be confused.*

*Comment: Line 499-512: please rewrite since it is not clear.*

**Response:** We apologize for any confusion caused. We have revised the discussion section in the revised manuscript to ensure clarity and accuracy.

*Comment: line 502, the authors give some explanations about the known function of PGS2 and PTS2 (Cox2). It should be done earlier, ideally in the result section.*

**Response:** We appreciate your insightful comment regarding the placement of explanations about the known functions of *COX-2* and PGE2.

Lines: 412-416, we have provided the explanation in the results section to enhance the clarity of our findings as follows:

“The gene encoding prostaglandin-endoperoxide synthase 2 (PTGS2), also known as cyclooxygenase-2 (COX-2), emerged as a compelling target for further investigation because its PGE2 product has been previously shown to reduce T cell proliferation and promote the activation of Tregs [Ref R19], observations that are consistent with our findings in MoDCs-P.”

[Ref R19] Sreeramkumar V, Fresno M, Cuesta N. Prostaglandin E2 and T cells: friends or foes? Immunol Cell Biol. 2012;90(6):579-86.

*Comment: line 159, misspelling: should be written “stained” instead of strained instead of*

**Response:** Thank you for the thorough review of the manuscript.

Lines: 195; we have written as follows:

“At 24 h post-infection, infected MoDCs (2×10^5^ cells) were stained with Zombie Red viability dye (BioLegend).”

*Comment: Line 549, Replace Th1 by Th2*

**Response:** Thank you for bringing up this important point. We have a reference to support the notion that a predominant IgG2 response in hamsters is indicative of Th1 immunity [Ref R20]. In our study, non-pathogenic strains appeared to promote the production of IFN-γ-producing CD4^+^ T cells, indicative of a Th1 response. Therefore, we have removed the section from the discussion section (line 544-551 in the original manuscript) that originally addressed the role of Th1 response to pathogenic leptospires, as it did not pertain to our findings on Th1 responses to non-pathogenic leptospires.

[Ref R20] Verma R, Joseph SK, Kushwaha V, Kumar V, Siddiqi MI, Vishwakarma P, et al. Cross-reactive molecules of human lymphatic filaria *Brugia malayi* inhibit *Leishmania donovani* infection in hamsters. Acta Trop (2015); 152:103–11.

*Comment: Lines 551-553, overstatement, TH1 response still needs to be investigated in leptospirosis*

**Response:** Thank you for bringing up this important point. We agree with the reviewer that we cannot conclude that Th1-type response is the key mediator of protective immunity against leptospirosis. We have revised this part as follows:

Lines: 558-561, “Therefore, our findings suggest, but do not definitely establish, that the induction of a Th1 response may be involved in the clearance of non-pathogenic leptospires. Additional studies are needed to elucidate the mechanisms responsible for their elimination.”

*Comment: In all the manuscript, figures legends and sup data, please write MoDCs are “infected” with Leptospira and keep the word “co-cultured” for co-culture experiments of MoDCs with CD4-T cells. It will greatly improve the understanding*.

**Response:** Thank you for your invaluable feedback. We appreciate your suggestion to use consistent terminology throughout the manuscript to enhance clarity. We have made the necessary adjustments and ensure that the term "infected" is consistently used to describe the interaction between MoDCs and *Leptospira*. Additionally, we will maintain the term "co-cultured" specifically for experiments involving the interaction between MoDCs and CD4^+^ T cells. This alignment in terminology will undoubtedly improve the overall understanding of our research.

*Comment: Fig 9 My suggestion would be show to only the panel D, to better recapitulate the work. Please better explain the Th1/Th2/ Treg phenotypes; also in text line 548-550 since it is very confusing.*
**Response:** Thank you for your thoughtful feedback regarding Fig 9. We agree with your suggestion and have shown only Panel D in Fig 7 in the revised manuscript to provide a clearer recapitulation of the work.

Lines: 364-366, we have explained the T cell phenotypes for clarity as follows:

“The expression of different cytokines and makers were selected to reflect the polarization state of activated CD4+ T cells as follows: IFN-γ for Th1 cells, IL-4 for Th2 cells, IL-17A for Th17 cells; and FoxP3 and IL-10 for Treg cells”.

Lines: 545-561, we have explained and rewritten the discussion section about the Th1 responses to non-pathogenic leptospires.

***As an editor mention:*** *Please, carefully address all the comments listed by the reviewers before submitting a revised version of your manuscript.*

**Response:** We have carefully addressed all comments from the reviewers, as outlined in the preceding responses. To clarify the modifications made to the manuscript, particularly the removal or merging of certain figures, we provide a summary of these changes below:

| **Figure number in the original manuscript** | **Figure in the original manuscript** | **Figure number in the revised manuscript** | **Figure in the revised manuscript** |
| --- | --- | --- | --- |
| Fig 1. MoDC maturation |  | Fig 1. MoDC maturation and cytokine production |  |
| Fig 2.  Cytokine production of infected MoDC |  | Fig 2. Apoptosis induction |  |
| Fig 3. The phagocytosis of leptospires by immature MoDCs |  | Fig 3. Intracellular cytokine production in CD4^+^ T cell by MoDCs-P and MoDCs-NP |  |
| Fig 4. Apoptosis induction in MoDCs-P and MoDCs-NP |  | Fig 4. Induction of CD4^+^ T cell proliferation by MoDCs-P and MoDCs-NP |  |
| Fig 5. Intracellular cytokine production (ICS) in CD4^+^ T cell by MoDCs-P and MoDCs-NP |  | Fig 5. DEG of MoDCs-P and MoDCs-NP |  |
| Fig 6. Induction of CD4^+^ T cell proliferation by MoDCs-P and MoDCs-NP |  | Fig 6. PGE2 level |  |
| Fig 7. DEG of MoDCs-P and MoDCs-NP |  | Fig 7. Schematic view of DC responses against pathogenic and non-pathogenic leptospires |  |
| Fig 8. PGE2 level |  |  |  |
| Fig 9. Schematic view of DC responses against pathogenic and non-pathogenic leptospires |  |  |  |

| **Supplementfigure number in the original manuscript** | **Supplement figure in the original manuscript** | **Figure number in the revised manuscript** | **Supplement figure in the revised manuscript** |
| --- | --- | --- | --- |
| S1 Fig.  Gating strategy and the percentage of cell purity |  | S1 Fig.  Gating strategy and the percentage of cell purity |  |
| S2 Fig. Representative histogram of the surface markers of MoDCs |  | S2 Fig. Representative histogram and FMO of the surface markers of MoDCs |  |
| S3 Fig. Phagocytosis of leptospires by MoDCs |  | S3 Fig. Fluorescence minus one of surface markers on MoDCs and isotype control of ICS-CD4^+^ T cells |  |
| S4 Fig. Percentage of viable MoDCs-P and MoDCs-NP |  | S4 Fig. Percentage of viable MoDCs-P and MoDCs-NP |  |
| S5 Fig. Representative scatter plots of apoptosis |  | S5 Fig. Representative of immunoblot analysis of cleaved caspase-3 at an MOI of 100 at 24 h |  |
| S6 Fig. The percentage of CD3^+^CD4^+^CD25^+^ cells and IL-2 production |  | S6 Fig. The percentage of CD3^+^CD4^+^CD25^+^ cells and IL-2 production |  |
| S7 Fig. Representative scatter plot and histogram of CD4^+^ cell proliferation |  |  |  |
