# Supplementary material for: Impaired functions of human monocyte-derived dendritic cells and induction of regulatory T cells by pathogenic Leptospira
Source: PLoS Negl Trop Dis. 2023 Nov 20;17(11):e0011781. doi: 10.1371/journal.pntd.0011781 (PMC10695387; doi:10.1371/journal.pntd.0011781)
Supplement: Attachment — Submitted filename: Response to reviewers.docx [file pntd.0011781.s009.docx]

Faculty of Medicine, Chulalongkorn University

1873 Rama IV Road, Pathumwan

Bangkok, 10330 THAILAND

[Kanitha.Pa@chula.ac.th](mailto:Kanitha.Pa@chula.ac.th); [Kpatarakul@gmail.con](mailto:Kpatarakul@gmail.con)

October 25, 2023

Dear Elsio Wunder Jr,

Thank you for providing us with the opportunity to revise our manuscript (Manuscript number: PNTD-D-23-00469) titled “Impaired Functions of Human Monocyte-Derived Dendritic Cells and Induction of Regulatory T cells by Pathogenic *Leptospira*”

We require your final decision regarding the conflicting opinions on statistical representation raised by reviewers #1 and #3. In our original manuscript, reviewer #1 commented that the letter-based system for indicating statistical differences was not obvious. Consequently, we revised all relevant figures to use a symbol-based system. However, in the revised manuscript, reviewer #3 expressed a preference for the original letter-based system, as detailed in our response to comment 2) from reviewer #3. Given the conflicting views of these two reviewers, we will adjust the statistical representation based on the editor’s guidance.

We greatly appreciate the invaluable suggestions and have made the necessary revisions based on the reviewers’ advice. Some comments were complex and contained multiple points. To address this, we have divided each complex comment into its individual components (italic and underlined) and responded in a point-by-point manner, as detailed below.

**Reviewer #1**

*Comment: I am happy that the authors addressed my concerns regarding the apoptosis assay by testing different MOIs and time points. The results of apoptosis are now much stronger and indisputable. However, the results showing that at 48h most MoDcs are dead makes me wonder if the authors should discuss in more detail this proportion of apoptotic cells in CD4 activation.*

**Response:** Thank you for your thoughtful suggestion. At 48 h post-infection, we observed that a high proportion of MoDCs infected with pathogenic leptospires underwent apoptosis, reaching nearly 80% for those infected with pathogenic *L. interrogans* serovar Pomona. These apoptotic MoDCs could be less effective as antigen-presenting cells, thereby potentially impairing the activation of CD4^+^ T cells. It is worth noting that, to investigate the CD4^+^ T cell responses in our study, we used MoDCs infected with leptospires at an MOI of 100 for 24 h. At this time point, cell viability was at least 80% for MoDCs infected with either pathogenic or non-pathogenic leptospires, as measured by the MTS assay (see S4 Fig.). This likely ensured a sufficient number of viable MoDCs for CD4^+^ T cell activation. Nonetheless, the mechanisms underlying MoDC apoptosis warrant further investigation in future studies.

Lines 536-542: We have addressed the impact of the high proportion of apoptotic MoDCs at 48 h post-infection on CD4^+^ T cell activation, as follows:

“We observed a statistically significant increase in MoDC apoptosis, especially in those infected with pathogenic *L. interrogans* for 48 h. These apoptotic MoDCs could be less effective as antigen-presenting cells, potentially impairing the activation of CD4^+^ T cells. Notably, for the assessment of the CD4^+^ T cell responses, we used MoDCs infected with leptospires at an MOI of 100 for 24 h. At this time point, cell viability was observed to be at least 80% in both MoDCs-P and MoDCs-NP, likely providing a sufficient number of MoDCs for CD4^+^ T cell activation.” For enhanced clarity, we have revised the manuscript as follows:

**Figure 3 legend:**

Lines 380-381, we have added “After 24 h of infecting MoDCs with leptospires”.

*The authors should also consider reviewing data from the literature that show the role played by PGE2 induced in and released by apoptotic cells that ultimately dampen immune responses (Hangai., et al, 2016) and, corroborate the author's findings.*

**Response:** We greatly appreciate your invaluable suggestion and the reference provided. According to the cited study by Hangai et al. (2016) [Ref R1], PGE2 induced in and released by dying cells, including apoptotic cells, serves as inhibitory damage-associated molecular patterns (iDAMPs). Therefore, the increase in apoptosis observed in MoDCs infected with pathogenic leptospires, as compared to those infected with non-pathogenic leptospires, could result in elevated levels of PGE2, thereby potentially suppressing immune responses and inflammation.

We have revised the Discussion section to include the following text:

Lines 523-530: “Furthermore, previous research indicates that PGE2 is induced in and released by dying cells, functioning as an inhibitory damage-associated molecular pattern (iDAMP) that suppresses immune responses and inflammation [Ref R1]. Therefore, it is possible that the observed higher levels of PGE2 secreted by MoDCs-P could result from increased apoptosis in these cells compared to MoDCs-NP. This elevated PGE2 level could then act as an iDAMP, thereby potentially impairing the responses of CD4^+^ T cells. However, further evaluation is needed to fully elucidate the role of the PTGS2/PGE2 pathway in both infected and apoptotic MoDC cell responses to leptospiral infection.”

[Ref R1]: Hangai S, Ao T, Kimura Y, Matsuki K, Kawamura T, Negishi H, Nishio J, Kodama T, Taniguchi T, Yanai H. PGE2 induced in and released by dying cells functions as an inhibitory DAMP. Proc Natl Acad Sci U S A. 2016 Apr 5;113(14):3844-9.

*Furthermore, the authors' schematic representation completely neglected the key fact that pathogenic strains induce apoptosis.*

**Response**: We have amended Fig 7 in the revised manuscript to include that pathogenic leptospires induced higher levels of apoptosis compared to non-pathogenic leptospires, as shown below.

**Reviewer #2:**

*Comment: The authors have made significant changes ad revised the manuscript. They have addressed most of the questions raised by the reviewers. The study is very thorough but major weakness of the study that it was performed in in vitro condition. Overall the study is significant and well executed.*

**Response:** Thank you for your constructive feedback, which has aiding in refining the quality and scientific rigor of our research. We acknowledge the major limitation that our study was conducted under *in vitro* conditions. However, we are committed to clearly stating both the strengths and weakness of our study.

**Reviewer #3:**

***Comment:*** *The authors carefully answered and addressed all comments which greatly improved this manuscript, that is now easy to read with clear messages. 
However, I still have 2 remarks*

*1) There is a major inconsistency about apoptosis and caspase 3 cleavage.* 
*At 48 hours after infection, the authors show by cytometry (with 3 dots corresponding to the 3 independent experiments with 3 different donors, nicely grouped) 80 % of apoptotic DC cells upon infection with pathogenic Leptospira Pomona, but the Western-Blots showing caspase 3 cleavage do not fully support this result. The WB in Fig2C is presented as a “representative Western blot …using samples from three donors to determine cleavage of caspase-3 in MoDCs. Then in, fig S5, a completely different WB (still labeled “representative” that I interpret as representative of the 2 other donors) shows that Leptospira strains (pathogenic and nonpathogenic) do not induce more cleavage of caspase 3 compared to the non-stimulated control. Therefore, the caspase 3 dependent apoptosis experiment is not convincing.*

**Response:** Thank you for your thorough review and invaluable feedback. We sincerely apologize for any confusion arising from the perceived inconsistency between Fig 2(C) and S5 Fig. Fig 2(C) serves as a representative result from three donors, showing the effects on MoDCs when infected with leptospires at an MOI of 100 for 48 h. This corresponds to the time when the highest proportions of apoptosis in MoDCs infected with pathogenic leptospires were observed, as demonstrated by flow cytometry in Fig 2(A) and 2(B). On the other hand, S5 Fig serves as a representative from three donors, demonstrating caspase-3 cleavage in MoDCs infected with leptospires at an MOI of 100 for 24 h, during which lower percentages of apoptotic cells were observed. Notably, in Fig 2(C) at 48 h post-infection, the band intensity of the 19-kDa cleaved caspase-3 was greater in the pathogenic group, especially *L. interrogans* serovar Pomona, compared to the non-pathogenic group, suggesting enhanced caspase-3 activation. In contrast, in S5 Fig no discernable difference in the band intensity of cleaved caspase-3 was observed among all strains 24 h post-infection. This observation may be attributed to the relatively low percentage of MoDC apoptosis at 24 h in both groups. To ensure the accuracy of our results, we used GAPDH as an internal control indicating a comparable number of cells across all samples. Therefore, the band intensity of cleaved caspase-3 in MoDCs infected with leptospires for 48 h and 24 h, as depicted in Fig 2(C) and S5 Fig respectively, clearly correlate with the proportion of apoptosis measured by flow cytometry in Fig 2(A) and 2(B). For enhanced clarity, we have revised the manuscript as follows:

**Figure 2 legend:**

Line 343: The title of Fig 2 has been revised to “Fig 2. Apoptosis induction in MoDCs-P and MoDCs-NP”.

Lines 346 and 350: Time points “for 24 and 48 h” have been added to Fig 2(A) and 2(B), respectively.

Lines 352-353: In Fig 2(C), the time point has been specified as “for 48 h” and “GAPDH served as an internal control.” has been added.

**S5 Figure legend:**

Lines 720-721 in the main text and Lines 44-45 in the supporting information: The title for S5 Fig has been revised for clarity to “S5 Fig. Representative Western blot analyses of cleaved caspases-3 in MoDCs-P and MoDCs-NP following infection at an MOI of 100 for 24 h.”, as shown below.

Line 48 in the supporting information: “GAPDH served as an internal control.” has been added.

*The authors should not crop the image of WB to show the upper non-cleaved band of pro-caspase 3, which is important to assess the efficiency of the positive control. The GAPDH controls can be put below.*

In response to the comment about showing the upper band of procaspase-3: our antibody is specific for the 17- and 19-kDa fragments of cleaved caspase-3 (Cell Signaling Technology, catalog number 9661). It does not recognize the full-length caspase-3 or procaspase-3 (approximately 37 kDa). Our goal was to detect the activation of caspase-3, represented by its cleavage, which confirms the apoptosis of MoDCs infected with leptospires. To ensure an accurate comparison of the band intensity of cleaved caspase-3 between samples, GAPDH (approximately 36 kDa) was used as an internal control on the same Western blot. Given the constraints of our antibodies and the protein quantity in our samples, we had to cut the membrane into 2 parts so that each part contained different molecular weight proteins of cleaved caspase-3 or GAPDH, and then incubate each part separately with antibodies against cleaved caspase-3 and GAPDH. We also used MoDCs treated with H_2_O_2_ as a positive control of caspase-3 cleavage in apoptotic cells. Therefore, the Western blots in Fig 2(C) and S5 Fig demonstrated only the cleaved caspase-3 and GAPDH bands, excluding procaspase-3. To enhance clarity, we have revised the manuscript as follows:

Lines 188-190: In the Methods section, we have added the following sentence: “The antibody specific for cleaved caspase-3 recognizes the 17- and 19-kDa fragments but does not cross-react with full-length caspase-3 or procaspase-3 (Cell Signaling Technology, catalog number 9661).”.

Lines 333-335: In the Results section, we have added the following text: “Immunoblot analysis using an antibody specific for cleaved caspase-3, which does not cross-react with procaspase-3, revealed the production of caspase-3 cleavage fragments at approximately 17 and 19 kDa.”.

In the revised manuscript, we have made changes to Fig 2(C) and S5 Fig, in which GAPDH bands were displayed underneath the bands for cleaved caspase-3, as shown below.

*The authors could have also provided the MTS assay at 48h in Fig S4.*

**Response**: To address the request for the inclusion of the MTS assay results at 48 h in S4 Fig, we would like to clarify that the primary objective of our MTS assay was to determine the viability of MoDCs infected with leptospires at an MOI of 100 for 24 h. MoDCs under these specific conditions were subsequently utilized to assess CD4^+^ T cell responses. We found that pathogenic leptospires induced a small decrease in cell viability (approximately 10-20%) in MoDCs after 24 h post-infection, as evidenced by the MTS assay shown in S4 Fig. In response to the reviewers’ comment on apoptosis in the original manuscript, we conducted apoptosis analyses at MOIs of 10, 50, and 100 for both 24 h and 48 h. These analyses revealed significant apoptosis, especially in MoDCs infected with *L. interrogans* serovar Pomona at an MOI of 100 for 48 h with approximately 80% apoptotic cells, as illustrated in Fig 2(B). Upon careful consideration, we opted not to perform the MTS assay for the 48-h time point. We are confident that our current presentation effectively conveys the key findings of our study. Including the MTS assay results at 48 h would neither substantially enhance our existing data nor alter the conclusions drawn from our findings.

*I think the data about apoptosis should be more robust (please add at least 5 more donors for WB and cytometry, before concluding of a strategy of leptospires to induce apoptosis to avoid the T-cell response.*

**Response:** We appreciate your focus on the robustness of the data and acknowledge the value of a larger sample size in enhancing the reliability of our findings. Initially, we utilized samples from five donors for most of the experiments presented in our original manuscript. In response to your comments regarding apoptosis, we repeated the apoptosis assays in the revised manuscript using three different MOIs and two different time intervals. Unfortunately, due to the extended storage duration of approximately one year, immature MoDCs in two out of the five original samples were insufficient in number and compromised in quality, rendering them unsuitable for further apoptosis studies. Consequently, we conducted the apoptosis assay with cells from the remaining three donors. We agree with your earlier observation that at 48 h after infection, our apoptosis results from flow cytometry were nicely grouped (with 3 dots corresponding to the 3 independent experiments with 3 different donors). Therefore, our results unambiguously indicated a significant difference in apoptosis levels between pathogenic and non-pathogenic leptospires.

Our research adheres to the institutional review board (IRB) guideline, which recommends using the minimal number of human samples required to achieve statistically significant results. While a larger sample size would indeed increase statistical power, our current data from three different donors already show a statistically significant difference between MoDCs infected with pathogenic and non-pathogenic leptospires. An amendment to the IRB protocol to include additional samples would necessitate a comprehensive reevaluation, which could be a time-consuming and cumbersome process. Therefore, we have opted not to extend the apoptosis assay to include more donors at this time.

*2) The change of statistical representation. I know that the statistics with R and letters seems at first difficult to understand, but it is indeed better to represent complete statistics and avoid fastidious representation with overlapping lines.*

**Response:** Thank you for your insightful suggestion regarding the use of a letter-based system for statistical representation. In the original manuscript, we received feedback from reviewer #1, who expressed concerns about the clarity of the letter-based system stating, “The letters system adopted by the authors to indicate statistical difference is not obvious.” Therefore, we transitioned to a symbol-based system in the revised manuscript to enhance clarity. Although the symbol-based system may result in overlapping lines and appear somewhat intricate, it is generally more conventional and widely recognized. Given the conflicting recommendations from reviewers, we will defer to the editor’s decision for the most appropriate statistical representation.

Thank you for the constructive feedback. We have addressed all comments and believe the revisions have strengthened our manuscript. We hope it will now meet the criteria for publication in your journal. We look forward to your decision and are prepared to make any further adjustments if required.

Best regards,

Associate Professor Kanitha Patarakul, MD, PhD
