# Supplementary material for: Impaired functions of human monocyte-derived dendritic cells and induction of regulatory T cells by pathogenic Leptospira
Source: PLoS Negl Trop Dis. 2023 Nov 20;17(11):e0011781. doi: 10.1371/journal.pntd.0011781 (PMC10695387; doi:10.1371/journal.pntd.0011781)
Supplement: Attachment — Submitted filename: Response to reviewers.docx [file pntd.0011781.s010.docx]

Faculty of Medicine, Chulalongkorn University

1873 Rama IV Road, Pathumwan

Bangkok, 10330 THAILAND

[Kanitha.Pa@chula.ac.th](mailto:Kanitha.Pa@chula.ac.th); [Kpatarakul@gmail.con](mailto:Kpatarakul@gmail.con)

November 7, 2023

Dear Elsio Wunder Jr,

Thank you for providing us with the opportunity to revise our manuscript (Manuscript number: PNTD-D-23-00469) titled “Impaired Functions of Human Monocyte-Derived Dendritic Cells and Induction of Regulatory T cells by Pathogenic *Leptospira*”

We greatly appreciate the invaluable suggestions and have made the necessary revisions.

***Comment:*** *These textual modifications should acknowledge all the limitations in the apoptosis experiments presented in the current study, with special emphasis on the small sample size used for these experiments.*

**Response:** Thank you for your suggestion regarding the limitations of the apoptosis experiment in our study. We have acknowledged these limitations, particularly the small sample size, and have updated the Discussion section accordingly:

Lines 558-564: “The limitations of the apoptosis experiment in the present study include a small sample size and the lack of an investigation into other cell death mechanisms. Increasing the sample size may strengthen the conclusions regarding the ability of pathogenic leptospires to induce apoptosis as a means to evade the CD4^+^ T cells response. To the authors’ knowledge, this is the first report to study apoptosis in human MoDCs induced by pathogenic leptospires through caspase-3 activation. Further research is necessary to gain a more comprehensive understanding.”

***Comment:*** *As for the statistical representation of significance, the Guest Editor prefers the "letter-based system," which was the Authors' initial choice and also seems to be preferred by Reviewer #3.*

**Response:** We appreciate the suggestion regarding statistical representation. In compliance, we have implemented the letter-based system in Figures 1, 2B, 3, 4B, 5B, and 6 of the revised manuscript. We have also made corresponding adjustments to S4 and S6 Figures in the supporting information. These figures are presented below.

Moreover, we have added the following explanatory text in the legends of each mentioned figure: “Conditions with distinct letters are significantly different (*P* < 0.05), whereas those with shared letters are not significantly different.”

For Fig 1, 2B, 3, 4B, 5B, and 6 in the main text, this text has been added to Lines 308-309, Lines 355-357, Lines 384-385, Lines 402-404, Lines 435-436, and Lines 461-462 respectively.

For S4 and S6 Figures in the supporting information, the text has been added to Lines 41-43 and Lines 64-65, respectively.

**Figure 1:**

**Figure 2:**

**Figure 3:**

**Figure 4:**

**Figure 5:**

**Figure 6:**

**S4 Figure:**

**S6 Figure:**

Thank you for your constructive feedback. We have addressed all comments and believe the revisions have strengthened our manuscript. We hope it will now meet the criteria for publication in your journal. We look forward to your decision and are prepared to make any further adjustments if required.

Best regards,

Associate Professor Kanitha Patarakul, MD, PhD+
